# General practitioner perceptions and experiences of managing perinatal mental health: a scoping review

**DOI:** 10.1186/s12884-023-06156-6

**Published:** 2023-12-02

**Authors:** Jacqueline Frayne, Sarah Seddon, Tamara Lebedevs, Talila Milroy, Beverly Teh, Thinh Nguyen

**Affiliations:** 1 Medical School, Discipline of General practice, The University of Western Australia, Crawley, Australia; 2 Pharmacy Department, Women’s and Newborn Health Service, King Edward Memorial Hospital, Subiaco, Australia; 3 South Metropolitan Health Services, Perth, Australia; 4 Peel and Rockingham Kwinana Mental Health Services, Rockingham, Australia; 5 Medical School, Discipline of Psychiatry, The University of Western Australia, Crawley, Australia

**Keywords:** General practice, Perinatal mental health, Scoping review, Psychotropic medication

## Abstract

**Background:**

General Practitioners (GPs) are involved in preconception, pregnancy, and postnatal care. Overall, mental health remains a significant contributor to disease burden affecting 1 in 4 pregnant women. Psychotropic medication prescribing occurs in almost 1 in 12 pregnancies, and appears to be increasing, along with the prevalence of mental health disorders in women of reproductive age. Perinatal mental health management is therefore not an unlikely scenario within their clinical practice. This scoping review aims to map current research related to GPs perceptions and experiences of managing perinatal mental health.

**Method:**

A comprehensive search strategy using nine electronic databases, and grey literature was undertaken between December 2021 and February 2023. Relevant studies were sourced from peer review databases using key terms related to perinatal mental health and general practitioners. Search results were screened on title, abstract and full text to assess those meeting inclusion criteria and relevance to the research question.

**Results:**

After screening, 16 articles were included in the scoping review. The majority focused on perinatal depression. Findings support that GPs express confidence with diagnosing perinatal depression but report issues of stigma navigating a diagnosis. Over the last two decades, prescribing confidence in perinatal mental health remains variable with concerns for the safety profile of medication, low level of confidence in providing information and a strong reliance on personal experience. Despite the establishment of perinatal guidelines by countries, the utilisation of these and other existing resources by GPs appears from current literature to be infrequent. Many challenges exist for GPs around time pressures, a lack of information and resources, and difficulty accessing referral to services.

**Conclusion:**

Recommendations following this scoping review include targeted perinatal education programs specific for GPs and embedded within training programs and the development of practice guidelines and resources specific to general practice that recognises time, services, and funding limitations. To achieve this future research is first needed on how guidelines and resources can be developed and best delivered to optimise GP engagement to improve knowledge and enhance patient care.

**Supplementary Information:**

The online version contains supplementary material available at 10.1186/s12884-023-06156-6.

## Introduction

Mental health conditions are a common presentation for women of reproductive age and occur in over a quarter of women during the perinatal period, defined as the period from conception, through pregnancy and the year after birth [1, 2]. General Practitioners (GPs) are involved in preconception, early pregnancy care and postnatal care. They also manage the majority of patients with mental health disorders, including high prevalence disorders such as anxiety and depression, commonly with prescribed antidepressant medications [3]. More and more, GPs also manage women with severe mental illnesses who may be planning to conceive or are pregnant as part of the shared care system which exists within our community mental health care system [4].

Almost 1 in 12 pregnancies are associated with psychotropic prescribing and evidence suggests that this rate is increasing [5, 6] alongside the prevalence of mental health disorders in women of reproductive age [7]. Estimates put around 15% of women of reproductive age in the United States (US) as being prescribed antidepressant medication [8] and combined with high rates of unplanned pregnancy in the general population rates of exposure to psychotropic medication may be higher than suggested. This makes reproductive planning and psychotropic medication counselling difficult in most cases, but vital when considering strategies to maximise mental wellbeing [9]. Dealing with the scenarios of mental health, psychotropic prescribing and pregnancy is therefore likely to be a frequent encounter within general practice and one that many GPs would be familiar with.

As part of the overall comprehensive care for pregnant women with existing mental illness, including identification, psychoeducation and referral for support counselling services, prescribing practices can have a significant impact, not just on the women’s mental health in terms of risk of relapse [10], but also on the potential risks to the pregnancy and the unborn child. It is a complex issue for women and health professionals. Available evidence from the United Kingdom suggests that many women ceased taking psychotropic medication when they learn they are pregnant [11]. Research in women with anxiety and depression and medication use, suggest that GPs have a strong influence on early decision-making [12]. Any discontinuation, switching, or lowering of doses of medication during planning or in the earlier stages of pregnancy needs to be carefully considered in the context of weighing up the risks and benefits of treatment, ideally as part of a shared decision-making process. This discussion is often led by the woman’s GP, however, there is a need to explore what advice GPs give and what sources of information they use to aid this process.

Clinical practice guidelines aim to reduce risk by outlining the research literature with the latest evidence and recommendations. Over the last three decades investment into research, education and raising awareness of perinatal mental health has occurred. Countries like Australia, the United Kingdom (UK) and United States have developed resources which focus on the area of perinatal mental health to assist community members and health professionals alike including clinical practice guidelines. Further, however we need to understand how these resources and clinical practice guidelines are used by general practitioners and how GPs can best be supported in our health system to facilitate best practice to improve health outcomes for women with mental illnesses and their babies.

A scoping review was deemed the most appropriate method of assessing the literature in trying to examine broadly General Practitioner experiences of managing perinatal mental health. Scoping reviews are often used to map existing research by synthesizing the evidence within a given field in terms of its nature, features, and volume and thereby identify gaps and make recommendations for future research [13]. This method is useful when the nature of the research question is complex or heterogenous.

## Method

Guidelines set out by Arksey and O’Malley [14] created the methodological framework for this review. To ensure a rigorous scoping review, five stages were adhered to: *identifying the research question, identifying relevant studies, study selection, charting the data*, and *collating, summarizing, and reporting the result.*

### Identifying the research question

Our aim was to broadly explore what is known about GPs’ experiences with perinatal mental health. Further, we wished to understand aspects related to management including clinical practice or guidelines, and psychotropic prescribing. We adopted a wide approach from the outset to gain a sense of the scope of literature and identify gaps in knowledge and further areas of research. As such, the research wanted to question *what is known from the existing literature of General practitioners’ (or equivalent) perceptions and experiences when managing perinatal mental health conditions.*

### Search Strategy

A comprehensive search strategy using nine electronic databases was undertaken between December 2021 and January 2022, with a final rerun of the search strategy completed in February 2023: GlobalHealth, PubMed, Informit Health Collection, EMBASE, MEDLINE, PsycINFO, Scopus, Best Practice, ClinicalKey; along with a search of alternative grey literature sources e.g., Google Scholar. No limits were set on date, language, or methodological design. The key words and derivatives were created by the authors and tailored for the specific requirements of each database (see Appendix A). Key terms were related to the perinatal period, psychotropic prescribing, best practice guidelines and mental health. The reference lists of included articles were also manually searched for any relevant research, as well as looking at articles which had cited the included articles.

### Citation management

All citations were imported into a software-based reference manager EndNote. Duplicates were removed with the aid of EndNote’s software, with further duplicates removed later in the review process.

### Eligibility criteria

All articles were screened in a two-stage process; titles and abstracts were screened for relevance, followed by a full-text review. If studies appeared to describe the management of mental health within the perinatal period, they were eligible for inclusion. Studies that were not written in English, and did not have an available translation, were excluded. Systematic reviews of perinatal mental health were excluded. Studies that appeared to assess general medication management within the perinatal period or general health within the perinatal period were excluded, along with studies that did not have a clear GP or equivalent role e.g., a family physician is the US equivalent of a GP. Studies that included other roles in addition to the GPs, such as psychiatrists, needed to provide a clear separation of findings to be included in the study, i.e., if researchers reported scores in aggregate, the article was excluded. Conference proceedings without full text articles were excluded. Articles that focused on the use of alternative or complementary medicines were also excluded. Article screening was completed by SS and reviewed by JF.

### Data charting

To complete a narrative review and synthesis of the articles, a data charting form was created in Excel to capture relevant information needed for analysis. Characteristics of the articles were extracted by SS and reviewed independently by JF. The information captured in the form is shown in Table 1.

**Table 1:** Studies included in qualitative synthesis

Author and year	Aim of the Study	Research Method	Location	Sample Size	Key Findings and Discussion
Bilszta et al. [15]	Explore primary health care physician’s beliefs and practices toward perinatal depression by investigating knowledge, attitudes, and practices affecting a physician’s decision to continue or discontinue antidepressant medication during pregnancy.	Quantitative: Survey	Australia and Canada	61 GPs; 33 Family Physicians	Age, years in practice, gender, or personal experiences with depression did not lead to a significant difference in treatment choice. Prescribing is a third line choice, after counselling or partner support. Physicians appeared more likely to taper medication in pregnant women, although this is linked to a six-fold increase in risk of relapse for women, indicating an incongruency between risk concerns and potential outcomes of medication management.
Brygger Venø et al. [28]	To explore GP’s perceived indicators of vulnerability among pregnant women in primary care.	Qualitative: Semi-structured focus groups	Southern Denmark	20 GPs	Patient doctor relationship is integral when deciding to ask further questions regarding mental health. GPs were aware of indicators of vulnerability to severe mental health but also referred to a *gut feeling* when picking up on intangible indicators.
Buist et al. [16]	To identify ways to improve detection of postnatal depression and access to treatment.	Quantitative: Survey vignette	Australia	246 GPs; 525 women	A difference between GP and patient preferences may lead to a hesitancy for women to bring up difficulties if they do not want to be prescribed medication. The length of time needed for a consult period when identifying postnatal depression is a significant time investment. Routine enquiry about women’s mood and coping is needed to identify women who might otherwise go undiagnosed.
Chew-Graham et al. [17]	To explore the views of GPs and health visitors on the diagnosis and management of postnatal depression.	Quantitative: Survey within a multicentre RCT	Various locations United Kingdom	19 GPs; 14 Health Visitors	GPs stress the importance of knowing the patient and using a psychosocial approach to making a diagnosis. GPs and Health Visitors agree that diagnosis of postnatal depression is important for management, but the ‘role’ of responsibility on detecting symptoms was unclear.
Chew-Graham et al. [18]	Exploration of views and attitudes of women and GPs following a disclosure of postnatal depression.	Qualitative: In-depth interviews within a multicentre RCT	Various locations United Kingdom	19 GPs; 14 Health Visitors; 28 Women	Psychosocial factors need to be addressed to diagnose and treat postnatal depression. Diagnoses, or pursuing a diagnosis, are avoided when health care providers feel they have nothing to offer e.g., continuity of care, referral to services. Questioning why a diagnosis of depression is not made by the healthcare professional, and suggesting training to address this issue, is too narrow an approach. Instead, a whole system approach is necessary to improve willingness to disclose, practitioner ability to listen and intervene, and a system that will facilitate management.
Edge [19]	To investigate health professionals’ views about perinatal mental healthcare for Black and minority ethnic women.	Qualitative: Interviews and Focus Groups	United Kingdom (Northern England)	42 health professionals (5 of which were GPs)	Physical health is often prioritised in the postnatal period, mental health can be overlooked. Services which limit continuity of care, health professional resistance to using psychometric tools, and lack of confidence in managing depression contribute to missed opportunities to detect and treat postnatal depression.
Glasser et al. [20]	To explore Israeli primary care physicians’ attitudes and practice regarding postpartum depression (PPD).	Quantitative: Three question survey	Israel	122 Paediatricians; 102 Family Practitioners	Most of the participants reported that it was important to recognize signs of postnatal depression and to act upon them. This resulted in most respondents reporting they would refer patients onward to mental health professionals. Significantly more family practitioners would screen for postnatal depression when compared to paediatricians.
Kean et al. [21]	To investigate current prescribing practices among GPs of antidepressants to women presenting in the first trimester of pregnancy and during breastfeeding.	Qualitative: Postal survey vignettes	United Kingdom (Scotland)	32 GPs	When given a list of potential medications to prescribe either in the first trimester, or during breastfeeding, GP prescribing patterns were inconsistent. Knowledge regarding classes of drugs was better than specific drugs within that class. Several GPs still had resistance to using medication for mothers with depression. There was also limited forward planning with prescribing medication that could be continued through until breastfeeding.
Khan [27]	Understand the role of GPs, and women’s experiences, in disclosure, identification and support with perinatal mental health	Mixed method: surveys and semi-structured interviews	United Kingdom (majority)	43 GPs for the survey; 3 GPs for the interview	Government action is needed to reduce the pressure on GPs and allow for longer consultations periods with women experiencing perinatal mental health problems. Higher education should work with RCGP Clinical Champion to support specific perinatal mental health training for qualified GPs. Local education and training boards should develop curriculum competencies relating to perinatal mental health through their GP training programmes. Evidence should be explored to assess the most effective way to use the six-week check to support mothers, babies, and families.
McCauley & Casson [22]	To develop an in-depth understanding of GPs’ experience of using guidelines in the treatment of perinatal depression and if this enabled them to empower women to become involved in treatment decisions.	Qualitative: Semi-structured interviews	United Kingdom (Northern Ireland)	8 GPs	The purpose of clinical guidelines is to enable GPs to empower women to make informed treatment decisions regarding pregnancy, however, the perceived usage of these guidelines is limited. GPs felt overwhelmed by too many guidelines, and conflicting safety data. GPs agree that involving women in the decision-making process is central to their empowerment, but this can be limited by the complexity of the presentation and to what level women want to be involved.
Mortimer et al. [29]	Investigate GPs’ and psychiatrists’ perceptions and experiences of caring for women with PTSD in the postnatal period.	Qualitative: Fictional case vignette	United Kingdom	6 GPs; 7 Psychiatrists	GPs can avoid diagnosis or attribute difficulties to other changes, often taking on a *watchful waiting* approach. Birth often isn’t considered a traumatic event by health care professionals, which potentially contributes to the way diagnoses are approached. Most training regarding postpartum diagnoses focuses on depression and psychosis, leading to a lack of training in other postpartum disorders such as post-traumatic stress disorder (PTSD).
Noonan et al. [30]	To explore GPs’ experiences of caring for women with perinatal mental health problems and their views on how best to prepare future GPs for a role in the provision of effective PMH care.	Qualitative: In-depth semi-structured interviews	Ireland	10 GPs	GPs described their multifaceted role in supporting women with perinatal mental health. GPs identified stigma, cultural and linguistic barriers to disclosure and care. Specialised care when indicated for women is limited due to under resourcing and long wait times. Training in perinatal mental health is needed, with some GPs suggesting a compulsory psychiatric rotation during training programs. Other options include e-modules to help build GP confidence and knowledge.
Santos Jr et al. [23]	To explore experiences of Brazilian physicians and nurses caring for women with postpartum depression in primary healthcare settings.	Qualitative: Open ended interviews	Brazil	10 nurses; 7 family health physicians	There appeared to be limited exposure to postpartum depression, which in turn created gaps in knowledge about postpartum depression, including its presentation and treatment. Limited clinical knowledge effects screening of postpartum depression and the documentation of symptoms. Gaps in GP training are highlighted as a potential causal factor for these issues.
Seehusen et al. [24]	To determine how frequently family physicians screen for PPD, what methods they use to screen, and what influences their screening frequency.	Quantitative: 25-item questionnaire	USA (Washington)	298 physicians	Screening for postpartum depression is not universal. When physicians do report screening for postpartum depression, they often don’t use a validated screening measure. Recency of training and sex differences are present when comparing those who screen more frequently, with more recent residency and female physicians screening more.
Ververs et al. [25]	To investigate where GPs and pharmacists in the Netherlands obtain information on the safety of gestational drug use and the pharmacotherapeutic approach when managing depression and anxiety during pregnancy.	Quantitative: Closed choice multiple choice questions	Netherlands	130 GPs; 144 pharmacists	Contraindications regarding safety of medication make it difficult and depend on differing sources of information. Differences in views on how to treat depression before, during and after pregnancy vary. GPs do consider the consequences of the mother’s illness outweighing the possible risks to the child. Concludes with the role of pharmacists being involved in developing clear policies and providing accessible information.
Williams et al. [26]	To explore the differences in the perception of teratogenicity risk of antidepressant and antianxiety medication commonly prescribed to pregnant women, medication counselling, prescribing practices, clinician resources and base knowledge of risk of antidepressant and antianxiety medications when used in pregnancy.	Qualitative: Survey	Australia	172 GPs; 373 Obstetrician/ Gynaecologists	GPs perceived higher rates of patient anxiety regarding anxiolytics and antidepressants, compared to obstetricians and gynaecologists. Both groups reported continued maternal concerns with fetal malformation due to medications. There is infrequent provision of written resources due to limited patient friendly resources. GPs often allotted more time in their consults to discussing medication risks and benefits. GPs saw themselves in a primary prescriber role, comparatively, and were less likely to refer to a mental health specialist. Both healthcare providers recommended a close patient-doctor relationship and clear communication when working with perinatal mental health and discussing medications. There is a modest interest in mental health disorders in pregnancy leading to a general lack of familiarity in the area and limited knowledge of the latest evidence.

*Note*: GPs = General Practitioners, USA = Unites States of America

From the information extracted, thematic process was adopted to highlight common themes within the articles included in the scoping review. These themes would then explain what the current landscape of GP experiences is when managing perinatal mental health, while also highlighting areas where there is a lack of research or limited understanding.

## Results

### Overview of the current literature

**Fig. 1:**
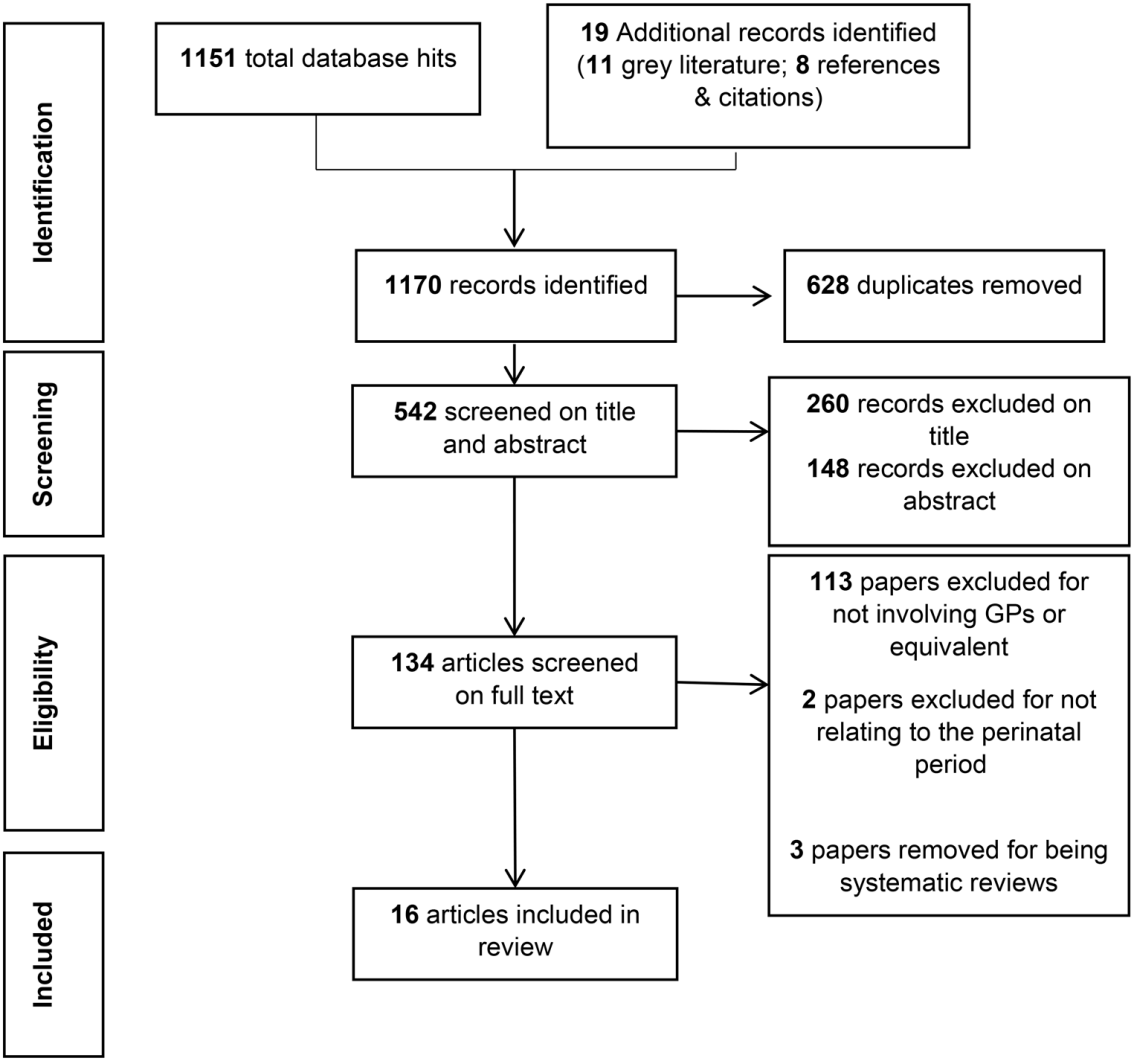
PRISMA flowchart for study selection process

The original search was conducted over December 2021 and January 2022 and yielded a total of 1,170 potential articles. After two-stage screening, 16 articles were included in the scoping review. The flow of articles from initial identification to inclusion are represented in Fig. 1.

Of these 16 included studies, 44% were carried out in the UK, followed by 18% in Australia, including one Australian and Canadian joint study. The final six studies were undertaken in six separate locations worldwide. Methodological designs consisted of qualitative, quantitative survey and mixed methods (see Table 1). No quality appraisal of the literature was undertaken in this review, only the extent and range of the literature is presented. To ensure all recent research was captured, the literature search was conducted again in February 2023; the search yielded no new articles which detailed GPs perspectives on prescription of psychotropic medication in the perinatal period.

All studies were required to assess the experiences and perceptions of general practitioners or equivalent. However, studies within the review also assessed the perceptions of health care visitors, obstetricians and gynaecologists, women, and psychiatrists. For the purposes of this review, the focus will remain on GP experiences and responses.

The majority of the articles focused on perinatal depression [15–25], depression and anxiety [26, 27], identifying psychosocial vulnerability [28], post-traumatic stress disorder [29] and overall perinatal mental health [30].

### Characterisation of general practitioner’s experiences

#### Identification and diagnosis

The literature supports findings that GPs appear confident in the diagnosis of mental health difficulties, with particularly awareness of postnatal/perinatal depression [16] rather than other less common diagnoses [29]. GPs acknowledge that perinatal mental health was an important area [24] though not without challenges. There was reported hesitancy for some GPs to diagnose due to perceived associations with ‘labelling and stigma’ [17, 18], women’s hesitancy in seeking help or disclosing issues again reported due to a perceived lack of acceptance of a problem, stigma and the interaction between the GP and patient [16, 18, 30], and inconsistencies of how symptoms are viewed between GPs and patients. This was particularly challenging in women from Culturally and Linguistically Diverse groups [19, 30].

A biopsychosocial approach to diagnosis was used most commonly by GPs, using perceived vulnerability and instinct, risk factors including social determinants, psychiatric and somatic conditions, supportive networks and inherent resilience [17, 28] with diagnosis conceptualised using this approach [18, 30]. Although GPs liked the idea of screening tools, there appeared to be a lack of use of these formal screening tools, such as the Edinburgh Postnatal Depression Scale (EPDS), to routinely screen women [17–19, 24]. These tools were seen as being more helpful in the postpartum period [24] and used more as an aid to diagnosis or as part of the referral process [30]. GPs felt that consistency of care was vital in supporting diagnosis [17–19, 26, 28–30] but also for management.

#### Pharmacological management

There was variability in GPs confidence in prescribing psychotropic medication. GP initiated antidepressant use in the perinatal period was high [26]. However, prescribing antidepressants as new onset in the perinatal period is viewed as second or third line in GPs, with psychological treatments and support structures taking precedence [15, 16]. This was potentially influenced by a lack of resources being available including access to care [16, 19, 21, 22, 30].

Decisions around treatment choice were influenced partially by patient factors [15] with a focus on patient centred care [30]. This was not without challenges as women were reported to stop medication abruptly on confirming a pregnancy [22]. There remained uncertainty around the safety profile of medication and low confidence in giving advice [15, 21, 22, 25, 26, 30]. Some GPs viewed treatment in pregnancy as very different compared to those who were not pregnant due to a perceived heightened vulnerability in pregnancy in general and concerns over legal liability regarding medication use in pregnancy [15, 30]. Inconsistent prescribing patterns [21, 25] were reported, with question concerning the undertreatment of women in the perinatal period [22]. The literature reported a strong reliance on GPs personal experience or experiences of colleagues in influencing their management [21, 22, 30] combined with a low use of guidelines [21, 22] to aid in prescribing practices.

#### Management resources

A limited knowledge of and access to educational opportunities, clinical exposure and appropriate resources impacted GPs in their practice [23, 26, 30]. Training was seen to increase the use of screening tools [24]. There was variable confidence in providing reliable information and resources to guide women in the decision making around these medications [22] and a lack of access of up to date safety data [25]. Pharmacy services were commonly used by GPs, as well as routine pharmacopeia’s, the internet [25] with often limited written information on medication provided [26], and then mainly sourced from MIMS and product information.

The limited use of guidelines corresponded to reports of GPs feeling overwhelmed by the quantity of guidelines for various medical conditions [22, 25], the lack of clear and specific direction [22, 30], often being too restrictive for individual circumstance [22], and too large in content to be useful [30].

#### Systems and service

Systemic limitations add difficulty. Many challenges exist for GPs around duration of consultations and how much can be covered. Perinatal mental health requires a significant time investment [16, 26] for adequate disclosure and consultation for both patients and clinicians [18]. The absence of clearly defined pathways [19] also impacts time commitment and the treatment plan. In some health systems it remains unclear where responsibility lies, and this can be compounded by issues around communication between services [20].

The overwhelming global systemic problem appears to be a lack of resources to refer patients on to [17, 19, 22, 30]. GPs generally lack trust in general psychiatric services, the prioritising of care and long wait times [29]. They would prefer to see local perinatal services [29] if they were readily available [30] but acknowledge that these services often have time limitations with care.

## Discussion

Our scoping review provides an overview of what is known in the existing literature on the perception and experiences of General Practitioners when managing perinatal mental health. Most studies focused on depression in the perinatal period with limited studies existing outside of depression and anxiety. The Royal Australian and New Zealand College of Psychiatrists [31] state that perinatal mental health includes all mental health disorders, recognising that the pregnancy and postnatal period provides a time of risk for new onset or relapse of symptoms. GPs are cognisant of the breadth of mental health disorders, with a recent Australian survey, Health of the Nation 2022 [32], stating psychological issues are one of the most common health conditions they manage.

In the literature GPs appear overall more confident in diagnosis than management, but areas of stigma for both patients and health professionals occur in disclosing and navigating a diagnosis. Two aspects discussed in this review include the use of a biopsychosocial approach and the importance of consistency of care, which are considered pillars of general practice and general practice education [33, 34]. The literature suggested that screening tools have their place in general practice but are possibly used more as an adjunct. It remains unclear if and how GPs are using these tools in their day-to-day practice currently given that the studies included in this review are over 10 years old and guidelines on screening have been introduced and updated subsequently. Consensus-based recommendations from the recently updated COPE perinatal management guidelines (2023) [35] recommend that all women should complete the EPDS preferably twice, in both the antenatal period and the postnatal period, ideally 6–12 weeks after the birth. In Australia, GPs in the vast majority are delivering postnatal care to women with potential barriers for screening existing including time limitations.

There appears to be support for using nonpharmacological therapies first line, with variation in medication prescription and use, including some strategies not supported by the evidence such as tapering of medication [15]. Delivering education is important, for both women, who were seen to cease medication abruptly on confirmation of pregnancy, consistent with studies in the literature [36], and for GPs who viewed management in pregnancy as distinct from those who are not pregnant and face uncertainty around the safety of medication. GPs disengagement with perinatal care in general is regarded as potentially leading to a deskilling in this area [27] and there are calls for a standardised GP trainee education [30] program specific to perinatal mental health.

The use of clinical or best practice guidelines in this area is reported as limited. Several countries have introduced guidelines and other resources for health professionals during the time of the studies included in this scoping review, but it is not known whether GPs are aware of, find benefit from and/or are using such resources. Clinical practice guidelines aim to reduce risk by guiding clinicians based on the research literature with the latest evidence and recommendations. A review of international clinical practice guidelines for perinatal depression, and antidepressant medication published by Molenaar et al. [3]. found the need for up-to-date and specific perinatal guidelines to help clinicians and patients in the shared decision-making process. We would propose that this needs to be specific and user friendly for GPs as they report feeling overwhelmed by the amount and volume of current guidelines for the many conditions that see within their practice.

The available literature suggests that GPs rely on personal experiences to guide them, with reliance on their own clinical experience and their colleagues. When considering GPs interaction with other health professionals, psychotropics are the leading medication class for which information on exposure during pregnancy and/or breastfeeding is requested from pharmacy services. This is in accordance with data published by other similar national and international medication information services, with requests increasing markedly since 2013 when the rate was 15.6% [37]. The consistent demand for these services despite the increasing availability of guidelines and web-based information indicates a need for clear and accessible information for prescribers, above what is provided by product information and drug categorisation in pregnancy [38], which these studies reported GPs used.

The lack of referral resources for ongoing management was a common theme in this review. A metanalysis by Ford et al. [39] in 2017 concludes that GPs remain frustrated by the lack of services and resources. It is uncertain whether the lack of resources is resulting in the undertreatment and underdiagnosis of women [17] or an increased use of prescription medication first line. Guidelines [35, 40] recommend seeking advice, preferably from a specialist in perinatal mental health particularly when initiating medication in pregnancy, but is this practical when resources are stretched? GPs are familiar with initiating pharmacological management with antidepressant and antianxiety medication [26] and would benefit from increased resources to support them in this role.

### Strengths and limitations

The strength of this review includes its systematic approach to identifying articles related to General Practitioners (or their equivalent) experiences around perinatal mental health more broadly and to map the research area. Limitations included the different health care services and resources available, and therefore variable experiences that occurred in differing countries. Further, the quality of literature was not appraised, therefore limiting the review to a descriptive account of GPs experiences.

### Conclusions and implications for research and practice

Overall, GPs feel somewhat confident in diagnosing mental health disorders within the perinatal period. They rely on a biopsychosocial and continuity of care model. Management of these conditions becomes more challenging with many depending on clinical experience and colleagues, with a lack of resources for consultations, referral, services, and information provision. Medication is frequently initiated by GPs with limited use of guidelines.

Recommendations following this scoping review include targeted perinatal education programs specific for GPs and embedded in training programs, and the development of practice guidelines specific to general practice that recognises time, services, and funding limitations. Future research identified include the use and value of screening tools in GP, and how guidelines and resources can be developed and best delivered to optimise GP engagement to improve knowledge and enhance patient care.

### Electronic supplementary material

Below is the link to the electronic supplementary material.

Supplementary Material 1: Appendix A

## Data Availability

All data generated or analysed during this study are included in this published article [and its supplementary information files].
