# Supplementary material for: General practitioner perceptions and experiences of managing perinatal mental health: a scoping review
Source: BMC Pregnancy Childbirth. 2023 Dec 2;23:832. doi: 10.1186/s12884-023-06156-6 (PMC10693076; doi:10.1186/s12884-023-06156-6)
Supplement: Supplementary file 1 — Supplementary Material 1: Appendix A [file 12884_2023_6156_MOESM1_ESM.docx]

**Appendix A: Database Search Terms**

| **Database** | **Search Terms** | **Date of Search** | **Total Number of Results** |
| --- | --- | --- | --- |
| Global Health | (Pregnan* OR prenatal OR Perinatal OR Antenatal OR maternal) AND (Psychotropic* OR medication OR drugs) AND (Guidelines OR protocols OR “practice guidelines” OR “Clinical practice guidelines” OR “evidence based practice”) AND (“mental health” OR “severe mental health” OR “mental illness” OR “severe mental illness”) [mp=abstract, title, original title, broad terms, heading words, identifiers, cabicodes] | 02.12.2021 | 8 |
|  |  | 13.02.2023 | 436 |
| PubMed | (((Pregnan*[Title/Abstract] OR prenatal[Title/Abstract] OR Perinatal[Title/Abstract] OR Antenatal[Title/Abstract] OR maternal[Title/Abstract]) AND (Psychotropic*[Title/Abstract] OR medication[Title/Abstract] OR drugs[Title/Abstract])) AND (Guidelines[Title/Abstract] OR protocols[Title/Abstract] OR "practice guidelines"[Title/Abstract] OR "Clinical practice guidelines"[Title/Abstract] OR "evidence based practice"[Title/Abstract])) AND ("mental health"[Title/Abstract] OR "severe mental health"[Title/Abstract] OR "mental illness"[Title/Abstract] OR "severe mental illness"[Title/Abstract]) | 02.12.2021 | 50 |
|  |  | 16.02.2023 | 58 |
| Informit Health Collection | [Abstract: pregnan* OR Abstract: prenatal OR Abstract: perinatal OR Abstract: antenatal OR Abstract: maternal] AND [Abstract: psychotropic* OR Abstract: medication OR Abstract: drugs] AND [Abstract: guidelines OR Abstract: protocols OR Abstract: 'practice guidelines' OR Abstract: 'clinical practice guidelines' OR Abstract: 'evidence based practice'] AND All Fields: abstract:( AND [All Fields: 'mental health' OR All Fields: 'severe mental health' OR All Fields: 'mental illness' OR All Fields: 'severe mental illness'] AND Limit To: Full Text | 06.12.2021 | 1 |
|  |  | 16.02.2023 | 0 |
| EMBASE | (Pregnan* or prenatal or perinatal or antenatal or maternal).ab. AND (Psychotrop* or medication or drugs).ab. AND (Guidelines or protocols or "clinical practice guidelines" or "evidence based practice").ab. AND ("mental health" or "severe mental health" or "mental illness" or "severe mental illness").ab. | 02.12.2021 | 96 |
|  |  | 16.02.2023 | 108 |
| MEDLINE | ((Pregnan* or prenatal or Perinatal or Antenatal or maternal) and (Psychotropic* or medication or drugs) and (Guidelines or protocols or practice guidelines or Clinical practice guidelines or evidence based practice) and (mental health or severe mental health or mental illness or severe mental illness)).ab. | 13.01.2022 | 46 |
|  |  | 16.02.2023 | 53 |
| PsycINFO | ((Pregnan* or prenatal or Perinatal or Antenatal or maternal) and (Psychotropic* or medication or drugs) and (Guidelines or protocols or practice guidelines or Clinical practice guidelines or evidence based practice) and (mental health or severe mental health or mental illness or severe mental illness)).ab. | 13.01.2022 | 33 |
|  |  | 16.02.2023 | 35 |
| Scopus | (TITLE-ABS-KEY (pregnan* OR prenatal OR perinatal OR antenatal OR maternal) AND TITLE-ABS-KEY (psychotropic* OR medication OR drugs) AND TITLE-ABS-KEY (guidelines OR protocols OR "practice guidelines" OR "Clinical practice guidelines" OR "evidence based practice") AND TITLE-ABS-KEY ("mental health" OR "severe mental health" OR "mental illness" OR "severe mental illness")) | 02.12.2021 | 298 |
|  |  | 16.02.2023 | 353 |
| Best practice | GP prescribing psychotropic medication in pregnancy | 06.01.2022 | 15 |
|  |  | 20.02.2023 | 17 |
|  | GP Prescribing psychotropic medication perinatal period | 06.01.2022 | 4 |
|  |  | 20.02.2023 | 5 |
| ClinicalKey | GP prescribing psychotropic medication in pregnancy | 06.01.2022 | 107 |
|  |  | 20.02.2023 | 141 |
|  | GP Prescribing psychotropic medication perinatal period | 06.01.2022 | 25 |
|  |  | 20.02.2023 | 30 |
| Alternative Sources (reference lists, Google Scholar, grey lit etc) |  | Ongoing | 27 |
| OneSearch UWA | GP prescribing psychotropic medication during pregnancy | 13.01.2022 | 411 |
|  |  | 16.02.2023 | 1 |
|  | GP prescribing psychotropic medication in perinatal period | 13.01.2022 | 104 |
|  |  | 16.02.2023 | 0 |
